# Predictors of Response and Mechanisms of Resistance to Antibody Drug Conjugates in Urothelial Carcinoma

**DOI:** 10.3390/curroncol33020103

**Published:** 2026-02-05

**Authors:** Jing Huang, Ademola Ojo, Bobby Liaw

**Affiliations:** Tisch Cancer Institute, Icahn School of Medicine at Mount Sinai, New York, NY 10029, USA

**Keywords:** antibody–drug conjugates, urothelial carcinoma, targeted therapy

## Abstract

Antibody–drug conjugates (ADCs) have reshaped the treatment landscape of urothelial carcinoma (UC) by enabling selective delivery of highly potent cytotoxic agents to tumor cells. Enfortumab vedotin, sacituzumab govitecan, and HER2-directed ADCs have demonstrated meaningful clinical activity across metastatic and earlier disease settings, with enfortumab vedotin plus pembrolizumab now established as a first-line standard of care. Despite these advances, therapeutic responses remain heterogeneous, and resistance frequently limits durability. This review summarizes current knowledge on predictors of response and mechanisms of resistance to ADCs in UC, highlighting the roles of target antigen expression and heterogeneity, genomic alterations, payload sensitivity, drug efflux transporters, and tumor microenvironmental factors. We discuss emerging biomarkers beyond antigen abundance, patterns of cross-resistance and treatment sequencing, and evolving strategies to overcome resistance, including next-generation ADC design and rational combination therapies. Advancing biomarker-driven patient selection and addressing mechanisms of resistance will be critical to maximizing the durability and clinical impact of ADCs in urothelial carcinoma.

## Simple Summary

Urothelial carcinoma, a common type of bladder cancer, has been difficult to treat once it spreads beyond the bladder. Antibody–drug conjugates are a newer form of cancer therapy that combine targeted antibodies with powerful cancer-killing drugs, allowing treatment to be delivered more directly to tumor cells. Several of these treatments have shown meaningful benefits for patients and are now used in both advanced and earlier stages of disease. However, not all patients respond well, and many cancers eventually stop responding to therapy. This review explains what is currently known about why these treatments work for some patients but not others, including differences in tumor biology, genetic changes, and the tumor environment. It also discusses new markers that may help predict response and future strategies to overcome resistance. These insights may guide future research and improve how treatments are selected and combined in clinical practice.

## 1. Introduction

Urothelial carcinoma (UC), the most common type of bladder cancer, accounts for approximately 90% of cases [1,2,3]. It is the sixth most common cancer in the United States, with a median age at diagnosis of around 70 years, occurring more frequently in males and in non-Hispanic Caucasian populations [4,5]. Tobacco use is the strongest risk factor, responsible for nearly half of all UC cases [6,7].

UC is characterized by one of the highest somatic mutation rates of any cancer, driving both inter- and intratumoral heterogeneity and clonal evolution across the urothelial lining. Key molecular features include mutations in tumor suppressors such as TP53 and RB1, common in muscle-invasive disease; FGFR3 alterations, often linked to non-muscle-invasive bladder cancer (NMIBC); and mutations in PI3K-AKT pathway components, including PIK3CA [1,2,4]. TERT promoter mutations represent one of the earliest and most frequent events, making them a potential biomarker for early detection [8].

Gene expression profiling has identified molecular subtypes of muscle-invasive bladder cancer (MIBC) with prognostic and therapeutic relevance, broadly categorized into luminal and basal groups [9,10]. In addition, UC includes less common histologic variants with aggressive potential, such as micropapillary, plasmacytoid, sarcomatoid, small cell/neuroendocrine, and nested forms [11]. These biologic and histologic differences may influence antigen expression patterns, treatment sensitivity, and the development of therapeutic resistance. Progression and therapeutic resistance in UC are also shaped by epithelial-to-mesenchymal transition (EMT) and tumor microenvironment (TME) factors that promote invasion, dissemination, and treatment escape [3,12].

The literature search strategy was a narrative review. We performed a non-systematic literature search using PubMed/MEDLINE, ClinicalTrials.gov, and major oncology meeting resources (including ESMO) to identify key studies evaluating antibody–drug conjugates in urothelial carcinoma, with emphasis on predictors of response and mechanisms of resistance. Searches included publications available through November 2025 and used terms such as “urothelial carcinoma,” “bladder cancer,” “antibody–drug conjugate,” “enfortumab vedotin,” “sacituzumab govitecan,” “trastuzumab deruxtecan,” “disitamab vedotin,” “Nectin-4,” “TROP-2,” “HER2,” “biomarker,” and “resistance.” We prioritized phase II/III clinical trials, guideline-informing studies, and selected translational/preclinical reports; seminal earlier studies were also included when relevant to ADC biology and resistance mechanisms.

## 2. Antibody Drug Conjugates

Antibody–drug conjugates (ADCs) pair a monoclonal antibody with a highly potent cytotoxic payload linked through a chemical connector, enabling targeted delivery of the drug to tumor cells while limiting systemic exposure [6].

**Figure 1:**
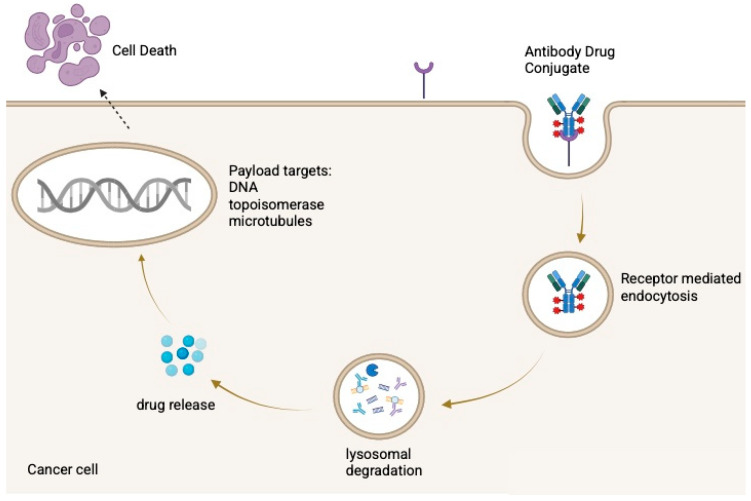
Mechanism of action of antibody–drug conjugates (ADCs). Created in BioRender. Huang, J. (2026) https://BioRender.com/3r97xz4.

After administration, the antibody binds its antigen, is internalized, and is trafficked to lysosomes, where the payload is released through enzymatic or acidic processing [13]. The liberated drug then disrupts cellular functions such as microtubule assembly or DNA integrity, inducing apoptosis as illustrated in Figure 1. Some ADCs also produce a bystander effect, in which the released payload diffuses into neighboring antigen-negative cells [6,14].

The therapeutic performance of ADCs is determined by three elements: the antibody, the linker, and the payload. The antibody provides tumor selectivity, typically using humanized or fully human IgG1 molecules for their long half-life, effector function, and efficient internalization [15]. Target antigens are chosen for high tumor expression and limited presence in normal tissues to reduce off-target effects. The linker bridges antibody to payload and must remain stable in circulation to prevent premature release but allow efficient liberation once inside the cell [6]. Cleavable linkers respond to intracellular cues such as low pH or enzymatic activity and can enable bystander killing, whereas non-cleavable linkers rely on antibody degradation, offering greater systemic stability but generally less bystander effect.

The payload is the core driver of ADC efficacy. Because only a small fraction of the administered drug reaches the tumor, payloads are designed to be extremely potent, often 100 to 1000 times stronger than conventional chemotherapy [14]. Most fall into two main categories: tubulin inhibitors which block microtubule assembly and disrupt cell division or DNA-damaging agents which cause DNA breaks or interfere with replication [13]. A key design parameter is the drug-to-antibody ratio (DAR), which defines the number of payload molecules per antibody. Higher DARs can enhance potency, but excessive loading may reduce stability and increase toxicity, necessitating careful optimization to maximize the therapeutic index [4].

ADCs consist of a monoclonal antibody linked to a cytotoxic payload via a cleavable or non-cleavable linker. After binding a tumor-associated antigen, the ADC is internalized and trafficked to lysosomes, where the payload is released through enzymatic, acidic, or redox-dependent processes. The liberated drug induces cell death, commonly through microtubule disruption (e.g., MMAE) or DNA damage via topoisomerase I (topo-I) inhibition (e.g., SN-38, DXd).

## 3. ADCs in Urothelial Carcinoma

For decades, management of locally advanced or metastatic UC relied largely on platinum-based chemotherapy and, more recently, immune checkpoint inhibitors (ICIs). ADCs now represent a major therapeutic pillar, functioning as “biological missiles” that selectively deliver highly potent cytotoxins to tumor cells by exploiting tumor-associated antigens [6,14,16]. This approach enhances efficacy while limiting systemic toxicity.

### 3.1. Targets

The clinical success of ADCs hinges on identifying tumor-associated antigens with high expression in cancer and minimal distribution in normal tissues. The most validated UC targets are Nectin-4 and TROP-2, with HER2 emerging as an additional biomarker-selected target [15,17].

- Nectin-4 (nectin cell adhesion molecule 4) is expressed in up to 87% of bladder cancer specimens, while remaining minimally detectable in normal adult tissues [15]. This tumor-selective expression makes Nectin-4 an appealing therapeutic target. It contributes to cell adhesion and tumor growth through PI3K–AKT signaling and undergoes rapid internalization upon antibody binding [13,15]. Enfortumab vedotin (EV), a Nectin-4-directed antibody–drug conjugate carrying monomethyl auristatin E (MMAE), leverages these properties and has become a key therapy in UC [18,19].
- TROP-2 (trophoblast cell-surface antigen 2), the target of sacituzumab govitecan (SG), is a transmembrane glycoprotein broadly overexpressed in epithelial malignancies, including UC, with minimal expression in normal tissues [17]. Its overexpression correlates with tumor growth and migration [17,20]. SG links an anti-TROP-2 antibody to SN-38, the active metabolite of irinotecan, which induces DNA strand breaks [17]. Although SG’s accelerated approval in metastatic UC was withdrawn after the phase III TROPiCS-04 trial failed to meet its primary overall-survival endpoint, the drug demonstrated antitumor activity, showing higher response rates than chemotherapy in TROPiCS-04 and comparable responses in TROPHY-U-01 study cohort 1, supporting continued interest in TROP-2 as a therapeutic target [20,21].
- HER2 (human epidermal growth factor receptor 2) is overexpressed in a subset of UC tumors, and higher expression has been associated with more aggressive biology and cisplatin resistance [17,20]. The advent of ADCs has renewed interest in HER2 targeting, as coupling HER2 binding with delivery of potent, membrane-permeable cytotoxic payloads has shown clinically meaningful activity in metastatic UC, including in the HER2-expressing cohort of the DESTINY-PanTumor02 trial, where response rates were higher in strongly HER2-positive tumors (IHC 3+) [18,20]. ADCs have also demonstrated activity in tumors with heterogeneous or low HER2 expression, as reported in a phase II study by Xu et al. (2022), whereas prior HER2-directed antibodies and tyrosine kinase inhibitors were largely ineffective [22].

### 3.2. FDA-Approved ADCs

Three ADCs have received FDA approvals relevant to UC [23,24], although SG subsequently withdrew its UC indication (Table 1). Historically, platinum-based regimens provided only modest improvements in overall survival (OS), with poor long-term outcomes and low five-year survival rates [25]. Earlier attempts to improve outcomes with chemo–immunotherapy combinations, such as in KEYNOTE-361 and IMvigor130, did not demonstrate significant OS benefit compared with standard platinum-based chemotherapy [26,27]. In 2023, the EV-302 trial demonstrated that EV plus pembrolizumab significantly improved progression-free survival (PFS) (12.5 vs. 6.3 months) and OS (31.5 vs. 16.1 months) compared with chemotherapy in the first-line setting [24,25]. Benefits were consistent across prespecified subgroups, including those defined by cisplatin eligibility and PD-L1 expression, indicating broad applicability. On the basis of these results, EV plus pembrolizumab is now established as a first-line standard of care in advanced disease. Mechanistically, EV targets Nectin-4 and delivers MMAE, while pembrolizumab restores antitumor immunity via PD-1 blockade, providing a rationale for combination therapy [25].

SG, the TROP-2 ADC, was approved in 2021 for patients previously treated with chemotherapy and immune checkpoint inhibitors (ICIs), but its indication was withdrawn in 2024 following the negative confirmatory TROPiCS-04 trial [21,28]. Accordingly, the discussion below highlights SG primarily for its mechanistic insights and lessons regarding Topo-I payload activity, toxicity mitigation, and resistance biology, rather than as a current guideline-endorsed standard option in routine practice. In the phase III TROPiCS-04 trial, SG achieved an objective response rate (ORR) of 23% in patients with advanced UC (aUC) previously treated with platinum-based chemotherapy and immune checkpoint inhibitors, consistent with phase II TROPHY-U-01 findings [21]. However, SG did not significantly improve OS or PFS compared with treatment of physician’s choice [28]. The absence of a survival advantage was largely attributed to early treatment-related toxicity. SG was associated with substantially higher rates of grade ≥ 3 adverse events and grade 5 events, primarily infection-related deaths occurring in the setting of neutropenia during the first treatment cycle. Notably, only 21% of patients received primary prophylaxis with granulocyte colony-stimulating factor (G-CSF), and none of the fatal neutropenic cases had received such prophylaxis [28]. These findings reflect the importance of supportive care optimization and careful patient selection for Topo-I–payload ADCs. Consistent with the withdrawal of the UC indication, SG has not been incorporated into major treatment guidelines (e.g., NCCN 2024–2025, ESMO) outside of clinical-trial settings.

Trastuzumab deruxtecan (T-DXd) received accelerated tumor-agnostic approval in 2024 for HER2 IHC 3+ solid tumors, including UC, based on DESTINY-PanTumor02 [19]. Efficacy was driven by HER2 expression rather than tumor type. Among 267 pretreated patients across seven tumor cohorts, including UC, T-DXd achieved a 37.1% ORR, with the greatest activity in the HER2 IHC 3+ subgroup. Median DOR, PFS, and OS were 11.3, 6.9, and 13.4 months, demonstrating durable benefit. These findings established HER2 expression as a meaningful therapeutic driver in UC and supported the FDA’s tumor-agnostic approval [29].

### 3.3. Perioperative and Intravesical Investigations

There is growing momentum to move ADCs into earlier-stage disease, particularly for patients who are cisplatin-ineligible and have limited neoadjuvant options. In cisplatin-ineligible MIBC, neoadjuvant EV achieved a 36% pathologic complete response (pCR) and 50% downstaging inEV-103 Cohorts H and J [18], while DV plus toripalimab demonstrated 63.6% pCR in HER2-positive disease [19]. Intravesical delivery approaches are also in development, including EV (EV-104) [30] and EpCAM-directed oportuzumab monatox with durvalumab for BCG-unresponsive NMIBC [31].

The phase III trial EV-303 (KEYNOTE-905) is directly testing perioperative benefit [18,19]. Early/interim analyses from the KEYNOTE-905 trial suggest that perioperative enfortumab vedotin plus pembrolizumab provides clinically meaningful and durable benefits for cisplatin-ineligible MIBC, a population historically lacking effective neoadjuvant options and experiencing poor outcomes with surgery alone. Interim/early analyses have reported high pathologic response rates, including a pathologic complete response rate of 57.1%, with event-free and overall survival medians not reached at the time of reporting [32]. Notably, the study population had a median age of 74 years, reflecting the older and more comorbid demographic commonly encountered among cisplatin-ineligible patients. Across available reports, perioperative feasibility appears preserved, with a manageable safety profile that did not preclude timely radical cystectomy in most patients, although continued monitoring remains essential. However, longer follow-up and full peer-reviewed reporting are required to confirm durability of benefit, characterize longer-term toxicity, and define the optimal role of this approach relative to existing standards. Collectively, these findings support continued investigation of ADC–ICI perioperative strategies in cisplatin-ineligible MIBC [32].

### 3.4. Ongoing Combination Trials

A broad array of ADC trials is ongoing, spanning monotherapy, combination, neoadjuvant, and tumor-agnostic strategies, and these studies are summarized in Table 2 below. For EV, current investigations include EV-103 across multiple cohorts, the phase III EV-302 trial, intravesical EV, and combinations with pembrolizumab, platinum agents, cabozantinib, erdafitinib, and durvalumab [18,19,24,30,33,34,35]. SG development is expanding as well, with TROPHY-U-01 Cohorts 3 to 6 [21,28]. Dual-ADC strategies using SG with EV and immunotherapy combinations with ipilimumab/nivolumab have shown encouraging early activity with objective response rates of 66 to 70% [36,37]. HER2-directed ADCs are also progressing, with DV plus toripalimab achieving high response rates in phase II studies and T-DXd demonstrating activity in both tumor-agnostic and UC-specific cohorts, although interstitial lung disease (ILD) and pneumonitis remain important safety considerations [19,36].

### 3.5. Overall Landscape

Overall, ADC development in UC is rapidly evolving, with EV-based regimens now incorporated into earlier lines of therapy and HER2-directed ADCs expanding treatment options for biomarker-selected patients. At the same time, multiple investigational ADCs targeting Nectin-4, TROP-2, B7-H3, SLITRK6, HER3, tissue factor (TF), and EpCAM are in clinical development. Despite these advances, response heterogeneity and acquired resistance remain common, underscoring the need to better define predictive biomarkers and resistance mechanisms to optimize patient selection and sequencing.

## 4. Predictors of Response to ADCs in Urothelial Cancers

Predicting response to ADCs in UC is complex and influenced by antigen expression, molecular alterations, resistance mechanisms, and broader aspects of tumor biology. Among these factors, antigen status remains the most reliable predictor, though additional genomic and immune-related features are increasingly recognized as important modifiers of outcome.

### 4.1. Enfortumab Vedotin

Nectin-4 is an important but imperfect biomarker for predicting response to EV. Higher levels of protein expression and the presence of Nectin-4 genomic amplification consistently correlate with improved clinical outcomes [4,13]. Tumors with high immunohistochemistry scores show longer PFS and higher response rates, and amplification is associated with particularly strong responses and improved survival [38,39,40]. In EV-302, patients with the highest expression levels experienced notably longer progression-free survival and response rates above 70 percent, reinforcing the association between target abundance and therapeutic effect [41]. However, EV also produces meaningful responses in tumors with low Nectin-4 expression, indicating that expression levels alone are insufficient as strict selection criteria [18,38]. This disconnect illustrates a broader challenge in ADC development, where biomarkers may demonstrate prognostic value but not reliably guide treatment decisions. Importantly, EV is currently administered without a required companion diagnostic, and Nectin-4 testing is not mandated for treatment selection.

The clinical utility of Nectin-4 is further limited by its biological heterogeneity. Expression often differs between primary and metastatic sites, with metastases frequently showing reduced membranous expression [42]. This variability may contribute to inconsistent responses within individual patients. Histologic variation presents additional challenges. Aggressive variants such as sarcomatoid and small-cell or neuroendocrine carcinomas generally exhibit very low or absent Nectin-4 expression, which likely accounts for their limited responsiveness to EV and supports the need for alternative therapeutic strategies in these populations [43]. Downregulation of Nectin-4 at disease progression is well documented and has been associated with acquired resistance, as discussed further in Section 5 [38,40]. These observations highlight several implications for future therapeutic approaches. Reassessing Nectin-4 expression at progression, rather than relying solely on baseline measurements, may help guide decisions about continued EV therapy or rechallenge.

### 4.2. Sacituzumab Govitecan

TROP-2 is broadly expressed in UC across RNA and protein levels, with lower expression seen primarily in neuroendocrine tumors [4]. Despite its ubiquity, TROP-2 levels have not correlated with outcomes in phase III studies, as demonstrated in TROPiCS-04, suggesting that baseline expression is necessary for activity but not predictive of efficacy [17,28]. By contrast, HER2-targeted ADCs such as DV and T-DXd show a stronger expression–response relationship, with the greatest activity in IHC 3+ disease. For example, T-DXd achieved an ORR of 56% in IHC 3+ tumors, while DV produced ORRs exceeding 70% in combination with immune checkpoint inhibitors [19,44].

Beyond antigen expression, specific genomic alterations appear to influence ADC outcomes. TP53 and MDM2 alterations have been associated with improved responses to both EV and SG, while methylthioadenosine phosphorylase (MTAP) deletions correlate with higher response rates to SG [42,43,45]. Conversely, KMT2D (a histone methyltransferase) alterations predict shorter PFS with EV plus pembrolizumab [46], and CDKN2B loss is enriched in primary refractory disease. Another analysis of the UNITE database identified alterations in ERBB2 and KDM6A, as well as high tumor mutational burden, as biomarkers predictive of better OS in patients who received EV [43,47]. Resistance to EV can arise from ABCB1/P-glycoprotein–mediated efflux of its MMAE payload, while secondary resistance often reflects loss of Nectin-4 expression in metastatic lesions [42].

Treatment sequencing also affects outcomes. SG shows reduced activity after EV, with real-world ORRs around 11 percent and median PFS of 2.1 months, although outcomes are somewhat better when SG is used immediately after EV [45,48]. Combination strategies are emerging as a critical approach. EV plus pembrolizumab has become a new first-line standard, and trials evaluating dual ADC regimens, such as EV plus SG, are ongoing. Toxicities may also provide biomarker insights; for example, EV-associated rash has been linked to improved outcomes, while SG toxicity risk is higher in patients with homozygous UGT1A1 variants, which reduce SN-38 glucuronidation and increase the risk of neutropenia and diarrhea [21,28].

## 5. Mechanisms of Resistance to Enfortumab Vedotin and Sacituzumab Govitecan

**Figure 2:**
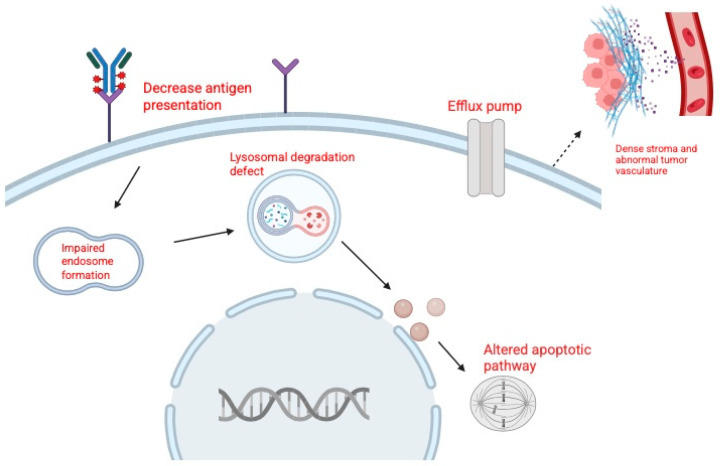
Mechanisms of resistance to antibody–drug conjugates (ADCs). Created in BioRender. Huang, J. (2026) https://BioRender.com/ua4uc51.

Resistance to ADCs such as EV and SG is a major challenge in the treatment of urothelial carcinoma. Tumor cells can escape ADC therapy through alterations in the target antigen, reduced sensitivity to the cytotoxic payload, changes in drug transport, or impaired intracellular processing (Figure 2). Broader factors, including tumor heterogeneity and the tumor microenvironment, also contribute to resistance [49].

Resistance to EV has been categorized into three main groups: target-related, payload-related, and drug efflux-related mechanisms [49,50,51]. Target-related resistance develops when tumor cells downregulate or lose Nectin-4, reducing ADC binding and uptake. Clinically, metastatic lesions have been shown to express less Nectin-4 than matched primary tumors, consistent with clonal selection of resistant populations [42,52]. Payload-related resistance arises when cells become less sensitive to MMAE, often through tubulin alterations that reduce drug binding. Preclinical studies support this mechanism, showing that EV-resistant bladder cancer cells are cross-resistant to MMAE itself [49]. Drug efflux-related resistance involves upregulation of ATP-binding cassette (ABC) transporters, particularly P glycoprotein (MDR1), which actively expels MMAE from tumor cells. Both clinical and preclinical data demonstrate higher MDR1 expression in resistant tumors, and inhibition of P glycoprotein has been shown to restore sensitivity to EV like ADCs [6,28,48].

In SN-38, similar to EV, SG resistance occurs through both target and payload mechanisms. Target-related resistance is associated with reduced TROP-2 expression, which may result from mutations or copy number loss, thereby limiting ADC binding [14]. Payload-related resistance develops when structural modifications in topoisomerase I impair SN-38 binding, reducing cytotoxicity [13].

In addition to drug-specific mechanisms, several general pathways undermine the activity of both EV and SG. Defective internalization or lysosomal dysfunction can prevent payload release, while inefficient trafficking may limit cytotoxic exposure. Tumor heterogeneity allows antigen-low or antigen-negative subclones to survive treatment, and features of the tumor microenvironment, such as dense stroma and abnormal vasculature, can act as physical barriers that restrict ADC penetration [4,49,50]. These multifactorial mechanisms highlight the complexity of resistance to ADC therapy in urothelial carcinoma.

Tumor resistance to ADCs can arise through reduced target recognition, altered intracellular processing, diminished sensitivity to the delivered payload, increased drug efflux, and microenvironmental or structural barriers that limit ADC penetration. These processes collectively decrease ADC efficacy and promote survival of resistant tumor cell populations.

## 6. Overcoming Resistance to ADCs

Although ADCs such as EV and SG have demonstrated substantial clinical benefit across multiple tumor types, acquired and intrinsic resistance remain major barriers to durable efficacy. Current research is focused on three complementary strategies: inhibiting tumor cell defense mechanisms, engineering next-generation ADC constructs, and employing rational combination therapies that target cancer through multiple pathways.

One of the best characterized resistance pathways involves the upregulation of efflux pumps, most notably P glycoprotein [6,42]. To address this, efflux pump inhibitors are being evaluated as co-treatments with ADCs. Preclinical models have shown that agents such as tariquidar can restore sensitivity to MMAE-based ADCs, leading to tumor regression in otherwise resistant models [4,13,51]. Early phase clinical studies with inhibitors such as cyclosporine have further validated this strategy, with response rates demonstrating the clinical feasibility of resensitizing tumors through modulation of efflux activity [15,42,51].

A second major avenue involves the rational redesign of ADC constructs to bypass resistance mechanisms. Because the three core components of an ADC, the antibody, linker, and payload, can be modified independently, this platform offers flexibility. Payload substitution is an important strategy; for example, switching from a tubulin inhibitor such as MMAE to a DNA damaging agent such as SN-38 can circumvent resistance linked to prior payload exposure [42]. Dual-payload ADCs, engineered to deliver two distinct cytotoxic agents, are also under development to address tumor heterogeneity and minimize the likelihood of single mechanism resistance [6]. In cases where resistance is driven by antigen downregulation, treatment may be switched to ADCs targeting alternative antigens. Furthermore, bispecific ADCs capable of binding to two distinct epitopes or targets have the potential to enhance tumor selectivity and reduce antigen escape [42]. Advances in molecular engineering have also yielded miniaturized conjugates, such as bicycle toxin conjugates (BTCs) [typically 9–20 amino acids, constrained in a rigid conformation] and antibody fragments, which improve tumor penetration and may overcome the physical limitations of larger ADCs in solid tumors [14]. In parallel, linker innovation remains a focus, with new designs aimed at enhancing plasma stability, resisting efflux, or exploiting novel cleavage mechanisms, including those activated by radiation or tumor-specific enzymes.

The third approach centers on rational combination therapy, which has shown great promise in both preclinical and clinical settings. Combining ADCs with agents that act through non-overlapping mechanisms can achieve synergistic effects and prevent resistance. A compelling example is the Dual Antibody Drug Conjugate (DAD) trial, which evaluated the combination of EV and SG in metastatic urothelial carcinoma [4,42]. By targeting two distinct antigens with different payloads, this strategy produced objective response rates significantly higher than either ADC alone, underscoring the therapeutic potential of combinatorial approaches. ADCs have also demonstrated synergy with ICIs. By inducing immunogenic cell death, ADCs increase tumor antigen presentation and enhance T cell-mediated antitumor responses, thereby complementing ICIs such as pembrolizumab [16,48]. This concept was validated in the landmark EV 302 trial, which established EV plus pembrolizumab as a first-line standard of care for advanced urothelial carcinoma. Looking forward, triplet regimens combining two ADCs with an ICI (such as the DAD IO trial) represent a promising frontier [42]. Beyond immunotherapy, ADCs are also being explored in combination with other targeted therapies, such as tyrosine kinase inhibitors, to simultaneously disrupt complementary oncogenic pathways and counteract resistance mechanisms [33,49].

In summary, resistance to ADCs is a multifactorial process involving drug efflux, target antigen modulation, limited tumor penetration, and intracellular processing barriers. Addressing these challenges requires a multipronged strategy that integrates pharmacological inhibition of resistance mechanisms, rational engineering of ADC constructs, and combinatorial regimens that leverage mechanistic synergy. Together, these efforts are shaping the next generation of ADC therapy, with the potential to extend durability of response, overcome resistance, and significantly improve clinical outcomes across multiple cancer types.

## 7. Future Directions and Conclusions

ADCs have transformed the management of UC, particularly in metastatic and treatment-refractory disease, and their role continues to expand as development moves into earlier disease settings, explores new targets, and advances conjugate engineering. Several major directions are likely to shape the next phase of ADC innovation in urothelial carcinoma.

A key area of progress is the continued move into earlier therapeutic settings. Agents such as EV and DV are being evaluated as neoadjuvant and adjuvant therapies for MIBC, including among cisplatin-ineligible patients. Trials such as KEYNOTE 905 (EV 303) and EV 103 (cohorts H and L) may help define how ADCs integrate into bladder sparing or perioperative strategies [53]. In parallel, intravesical ADC delivery is under investigation in NMIBC [31], potentially offering new bladder-preserving options for patients with BCG-unresponsive disease who currently have limited alternatives.

Combination therapy represents another central developmental pathway. Pairing ADCs with ICIs has already become a standard approach in mUC but combinations are now being tested in earlier stage and perioperative settings, leveraging ADC-induced immunogenic cell death to enhance immune responses. Additional rational combinations, including ADCs with TKIs such as tucatinib [54], anti-angiogenic therapies, or even dual or serial ADC regimens, aim to overcome resistance mechanisms and amplify therapeutic efficacy. Meanwhile, ADC engineering is rapidly evolving. Next-generation constructs featuring site specific conjugation, optimized drug to antibody ratios, and improved linker stability are designed to enhance pharmacokinetics, potency, and safety. Novel payload classes, including topoisomerase inhibitors, immunostimulatory agents, and protein degraders, along with increasingly tumor-selective linkers, are under development to reduce off-target toxicity [17,55]. Dual-payload and bispecific ADCs capable of delivering two cytotoxins or binding multiple antigens are emerging as promising strategies to address tumor heterogeneity and mitigate resistance [56].

Target expansion and the emergence of new conjugate platforms are rapidly broadening the therapeutic scope of ADCs in urothelial carcinoma. Beyond established targets such as Nectin-4 and TROP-2, interest is increasing in HER2, B7 H3, HER3, and EpCAM [56,57,58], with agents such as disitamab vedotin and oportuzumab monatox showing encouraging early activity. Multiple new Nectin-4-directed ADCs, including LY4101174, CRB 701, and 9MW2821, are advancing through early phase evaluation [7,59,60]. TROP-2 remains a major focus, with next-generation candidates such as sacituzumab tirumotecan and datopotamab deruxtecan progressing in clinical development [33,56,57]. The HER2-targeted space is likewise diversifying, as illustrated by sirtratumab vedotin, ifinatamab deruxtecan, and novel constructs such as MRG002 [30,31,34]. At the same time, innovative conjugate platforms are extending beyond traditional ADC designs. Bicycle toxin conjugates such as BT8009 [61], which use compact peptide scaffolds to enhance tumor penetration and reduce systemic exposure, are now in phase II and III testing and may ultimately complement or surpass conventional ADCs in selected settings [55]. A list of next-generation and novel ADCs can be found in Table 3.

Over the next 3–5 years, biomarker-driven ADC selection in UC will most realistically enter routine practice through standardized HER2 testing to identify patients eligible for HER2-directed ADCs. In contrast, enfortumab vedotin is currently administered without a required selection biomarker, and Nectin-4 testing is more likely to remain exploratory unless prospectively validated. Key barriers include intrapatient antigen heterogeneity, dynamic antigen loss under treatment pressure, limited access to metastatic tissue, lack of harmonized assays and scoring systems, and practical issues such as turnaround time and reimbursement. Embedding biomarker analyses into pivotal trials and incorporating liquid biopsy and spatial profiling approaches may help enable more individualized ADC selection and sequencing.

In summary, ADCs play an increasingly central role across the full spectrum of urothelial carcinoma. Ongoing progress in target identification, conjugate design, biomarker refinement, and resistance management is steadily moving the field toward more precise and durable treatment approaches. As clinical data continue to mature, these agents have the potential to reshape standard of care in both early and advanced disease, providing more meaningful and sustained benefit for a wider range of patients.

## Figures and Tables

**Table 1:** Summary of key ADCs in urothelial carcinoma.

Drug	Target	Payload	Trial/Phase	Key Outcomes
Enfortumab vedotin	Nectin-4	MMAE	EV-301 (III)	Improved OS, ORR ~40%
Enfortumab vedotin + Pembrolizumab	Nectin-4	MMAE	EV-302 (III)	Established first-line SOC
Sacituzumab govitecan	TROP-2	SN-38	TROPHY-U-01 (II)	ORR ~27%
Sacituzumab govitecan	TROP-2	SN-38	TROPiCS-04 (III)	Confirmatory phase III outcomes
Trastuzumab deruxtecan	HER2	Deruxtecan	Early-phase cohorts	Early efficacy signals

**Table 2:** Current ongoing trials of ADCs in urothelial carcinoma.

ADC/Agent	Study/Trial	Setting/Population	Regimen	Key Outcomes or Notes
Enfortumab vedotin (EV)	EV-103 Cohort H, L (NCT03288545)	Neoadjuvant/perioperative cisplatin-ineligible MIBC	EV monotherapy	Feasibility, pathologic response, and safety of EV as perioperative therapy in cisplatin-ineligible MIBC ^1^
	EV-302 (phase III) (NCT04223856)	Previously untreated locally advanced/metastatic UC (la/mUC)	EV ± pembrolizumab vs. platinum-based chemotherapy	Confirmatory first-line trial; EV + pembrolizumab now a guideline-endorsed standard ^2^
	EV-104 (NCT05014139)	Non-muscle-invasive bladder cancer (NMIBC)	Intravesical EV	First-in-human intravesical EV; evaluates local delivery, safety, PK ^3^
	NCT04878029	Advanced/metastatic UC	EV + cabozantinib	Combines EV with a multi-TKI; explores synergy via anti-angiogenic modulation ^4^
	NCT04963153	Advanced/metastatic UC	EV + erdafitinib	Targets a genomically defined subgroup (FGFR3); dual-target strategy ^5^
	EV-103 Cohorts A, B, J, K (NCT03288545)	Locally advanced/metastatic UC	EV + pembrolizumab	High objective response rates (ORR); ongoing durability and safety evaluation ^6^
	EV-103 Cohort D (NCT03288545)	Locally advanced/metastatic UC	EV + cisplatin	Tests whether adding EV to standard cisplatin improves response vs. chemotherapy alone ^7^
	EV-103 Cohort E (NCT03288545)	Locally advanced/metastatic UC	EV + carboplatin	Analogous to Cohort D for carboplatin-eligible patients ^7^
	EV-103 Cohort G (NCT03288545)	Locally advanced/metastatic UC	EV + platinum + pembrolizumab	Triplet regimen designed to maximize first-line efficacy; safety/feasibility being defined ^7^
Nectin-4/HER2-directed combos	VOLGA trial (NCT04960709)	Neoadjuvant/perioperative MIBC, cisplatin-ineligible	EV + durvalumab (±tremelimumab)	Perioperative chemo-free immunotherapy/ADC strategy ^8^
Sacituzumab govitecan (SG)	TROPHY-U-01 Cohorts 3–6 (NCT03547973)	Advanced/metastatic UC (platinum-refractory, cisplatin-ineligible, maintenance, first-line ICI)	SG ± pembrolizumab, cisplatin, or maintenance ICI	Multi-cohort program evaluating SG alone and in combinations across treatment lines ^9^
	DAD trial (NCT04724018)	Heavily pretreated advanced/metastatic UC	Dual SG + EV	Early ORR ≈ 70% in expansion cohorts; explores dual-ADC strategy in a high-unmet-need setting ^10^
	NCT04863885	Cisplatin-ineligible UC	SG + ipilimumab/nivolumab	Dual checkpoint-inhibitor + ADC; early ORR ≈ 66.6% ^11^
HER2-directed ADCs	RC48-C014 (DV + toripalimab) (NCT04264936)	HER2-positive UC	DV + toripalimab (anti-PD-1)	ORR 71.8–100% in early cohorts; strong activity even in HER2-low tumors ^12^
	RC48-C016 (NCT05302284), DV-001 (NCT05911295)	HER2-positive UC	DV ± pembrolizumab vs. chemotherapy	Ongoing randomized trials to define DV-based regimens against standard chemotherapy ^13^

^1^ EV-103 Cohorts H and L (NCT03288545). ^2^ EV-302 (NCT04223856); Powles, T. et al., *N. Engl. J. Med.*, 2024 [25]. ^3^ EV-104 (NCT05014139). ^4^ Enfortumab vedotin plus cabozantinib (NCT04878029). ^5^ Enfortumab vedotin plus erdafitinib (NCT04963153). ^6^ EV-103 Cohorts A, B, J, and K (NCT03288545). ^7^ EV-103 Cohorts D, E, and G (NCT03288545). ^8^ VOLGA (NCT04960709). ^9^ TROPHY-U-01 Cohorts 3–6 (NCT03547973). ^10^ DAD (NCT04724018). ^11^ Sacituzumab govitecan plus ipilimumab and nivolumab (NCT04863885). ^12^ RC48-C014 (NCT04264936). ^13^ RC48-C016 (NCT05302284); DV-001 (NCT05911295).

**Table 3:** Next-generation antibody–drug conjugates (ADCs) and novel targets in urothelial carcinoma.

Target/ Pathway	Agent	Platform/ Payload	Trial (Phase)	Key Clinical or Translational Features
Nectin-4	LY4101174 (ETx-22) [62]	ADC	NCT06238479 (Early phase)	Next-generation Nectin-4-directed ADC designed as a potential alternative to enfortumab vedotin; early clinical evaluation ongoing
	CRB-701 (SYS6002) [63]	ADC	NCT06265727 (Phase I)	No dose-limiting toxicities observed; partial responses across multiple dose levels; engineered with optimized linker–payload to improve safety
	9MW2821 [64]	ADC	NCT05216965 (Phase I/IIa)	Investigational Nectin-4 ADC evaluated across advanced solid tumors including UC; UC-specific efficacy data pending
	BT8009 [61]	Bicycle toxin conjugate (BTC; MMAE)	NCT04561362 (Phase II/III)	Small bicyclic peptide scaffold enabling rapid tumor penetration and renal clearance; ORR 50% and DCR 75% in early la/mUC cohorts
TROP-2	Sacituzumab tirumotecan (sac-TMT; MK-2870/SKB264) [65]	ADC (Topo-I inhibitor)	KEYMAKER-U04/NCT04152499 (Phase I/II)	Confirmed ORR 45.5% (2L) and 26.3% (≥3L); median OS 11.5 months in later-line UC; grade ≥3 AEs in ~59%, mainly hematologic
	Datopotamab deruxtecan (Dato-DXd) [66,67]	ADC (DXd payload)	TROPION-PanTumor03/NCT05489211 (Phase II)	Combination with rilvegostomig yielded ORR 68.2% (1L) and 33.3% (2L); high disease control rates
	BAT8008 [68]	ADC (Topo-I inhibitor)	NCT05620017 (Phase I)	Demonstrated tolerable safety; proof-of-concept activity in non-UC cohorts supports further UC exploration
	OBI-992 [69,70]	ADC (Exatecan-based)	NCT06480240 (Phase I)	Retained activity in P-gp/BCRP-overexpressing models, suggesting reduced susceptibility to multidrug resistance
	BHV-1510	ADC	NCT06384807 (Phase I)	Early-phase TROP-2 ADC under clinical evaluation; efficacy data not yet reported
	FDA018-ADC	ADC	NCT05174637 (Phase I)	Early-phase TROP-2 ADC reflecting continued diversification of linker–payload strategies
HER2/Related Pathways	Sirtratumab vedotin (AGS15E) [71]	ADC (MMAE; SLITRK6 target)	NCT01963052 (Phase I)	ORR 18.3% overall (35.7% at RP2D); ORR 27.3% in CPI-exposed patients; median PFS 16 weeks
	Ifinatamab deruxtecan [72]	ADC (DXd; B7-H3 target)	IDeate-PanTumor02/NCT0633006 (Early phase)	High-DAR, cleavable linker DXd ADC; UC-specific outcomes pending
	MRG002 [73]	ADC (MMAE; HER2)	NCT04839510 (Phase II)	ORR 65% (9% CR) and DCR 91% in HER2-positive la/mUC, indicating strong HER2-directed activity
Other Emerging Targets	BL-B01D1 [58]	Bispecific ADC (EGFR/HER3; Topo-I payload)	NCT05785039 (Early phase)	First-in-class bispecific ADC; high response rates in EGFR-mutant NSCLC; UC cohorts ongoing

ADC = antibody–drug conjugate; BTC = bicycle toxin conjugate; CPI = checkpoint inhibitors; UC = urothelial carcinoma; la/mUC = locally advanced or metastatic urothelial carcinoma; ORR = objective response rate; DCR = disease control rate; OS = overall survival; RP2D = recommended phase II dose; MMAE = monomethyl auristatin E; DXd = deruxtecan; Topo-I = topoisomerase I inhibitor. Reported efficacy reflects available UC cohorts when specified; some trials enrolled pan-tumor populations.

## Data Availability

No new data were created or analyzed in this study. Data sharing is not applicable to this article.

## Abbreviations

ABCB1	ATP-binding cassette subfamily B member 1 (P-glycoprotein/MDR1)
ADCs	Antibody–drug conjugates
AKT	Protein kinase B
aUC	Advanced urothelial carcinoma
BCG	Bacillus Calmette–Guérin
BTC	Bicycle toxin conjugate
CPI	Checkpoint inhibitor
CR	Complete response
DAD	Dual antibody–drug conjugate
DAR	Drug-to-antibody ratio
DCR	Disease control rate
Dato-DXd	Datopotamab deruxtecan
DESTINY-PanTumor02	Study name (trastuzumab deruxtecan tumor-agnostic trial)
DNA	Deoxyribonucleic acid
DV	Disitamab vedotin
EMT	Epithelial–mesenchymal transition
EpCAM	Epithelial cell adhesion molecule
ERBB2	Gene encoding HER2
ESMO	European Society for Medical Oncology
EV	Enfortumab vedotin
EV+P	Enfortumab vedotin plus pembrolizumab
FGFR	Fibroblast growth factor receptor
FGFR2/3	Fibroblast growth factor receptor 2/3
G-CSF	Granulocyte colony-stimulating factor
HER2	Human epidermal growth factor receptor 2
HER3	Human epidermal growth factor receptor 3
ICI	Immune checkpoint inhibitor
IHC	Immunohistochemistry
ILD	Interstitial lung disease
la/mUC	Locally advanced or metastatic urothelial carcinoma
MDR1	Multidrug resistance protein 1 (P-glycoprotein)
MIBC	Muscle-invasive bladder cancer
MMAE	Monomethyl auristatin E
MTAP	Methylthioadenosine phosphorylase
mUC	Metastatic urothelial carcinoma
NCCN	National Comprehensive Cancer Network
NMIBC	Non-muscle-invasive bladder cancer
ORR	Objective response rate
OS	Overall survival
pCR	Pathologic complete response
PD-1	Programmed cell death protein 1
PD-L1	Programmed death-ligand 1
PFS	Progression-free survival
PI3K	Phosphoinositide 3-kinase
PK	Pharmacokinetics
RNA	Ribonucleic acid
RP2D	Recommended phase II dose
SG	Sacituzumab govitecan
SN-38	Active metabolite of irinotecan
SOC	Standard of care
T-DXd	Trastuzumab deruxtecan
TF	Tissue factor
TKI	Tyrosine kinase inhibitor
TME	Tumor microenvironment
Topo-I	Topoisomerase I
TROP-2	Trophoblast cell-surface antigen 2
UC	Urothelial carcinoma
